# Optimising Non-Patterned MoO_3_/Ag/MoO_3_ Anode for High-Performance Semi-Transparent Organic Solar Cells towards Window Applications

**DOI:** 10.3390/nano10091759

**Published:** 2020-09-06

**Authors:** Lichun Chang, Leiping Duan, Ming Sheng, Jun Yuan, Haimang Yi, Yingping Zou, Ashraf Uddin

**Affiliations:** 1 School of Photovoltaic and Renewable Energy Engineering, University of New South Wales, Sydney, NSW 2052, Australia; li-chun.chang@student.unsw.edu.au (L.C.); ming.sheng@student.unsw.edu.au (M.S.); haimang.yi@unsw.edu.au (H.Y.); 2 College of Chemistry and Chemical Engineering, Central South University, Changsha 410083, China; junyuan@csu.edu.cn

**Keywords:** semi-transparent, organic solar cells, colour perception, transparent electrodes, non-fullerene

## Abstract

Semi-transparent organic solar cells (ST-OSCs) have attracted significant research attention, as they have strong potential to be applied in automobiles and buildings. For ST-OSCs, the transparent top electrode is an indispensable component, where the dielectric/metal/dielectric (D/M/D) structured electrode displayed a promising future due to its simplicity in the fabrication. In this work, by using the MoO_3_-/Ag-/MoO_3_-based D/M/D transparent electrode, we fabricated ST-OSCs based on the PM6:N3 active layer for the first time. In the device fabrication, the D/M/D transparent electrode was optimised by varying the thickness of the outer MoO_3_ layer. As a result, we found that increasing the thickness of the outer MoO_3_ layer can increase the average visible transmittance (AVT) but decrease the power conversion efficiency (PCE) of the device. The outer MoO_3_ layer with a 10 nm thickness was found as the optimum case, where its corresponding device showed the PCE of 9.18% with a high AVT of 28.94%. Moreover, the colour perception of fabricated ST-OSCs was investigated. All semi-transparent devices exhibited a neutral colour perception with a high colour rendering index (CRI) over 90, showing great potential for the window application.

## 1. Introduction

Organic solar cells (OSCs) have the benefits of inexpensive fabrication, flexibility, lightweight, and nature of semitransparency [1,2,3]. Recently, OSCs have attracted significant research attention, and many researchers have been devoted to improving the power conversion efficiency (PCE) and stability of the device [4,5,6,7]. A short time ago, the PCE of the single-junction and tandem OSCs had recorded 18.22% and 17.36%, respectively [8,9]. Due to the semi-transparent (ST) characteristic, OSCs show potential in the application for automobiles or buildings to generate power as windows or curtains [10,11,12,13,14]. Especially in the building industry, the building-integrated photovoltaic (BIPV) technology is growingly becoming one of the efficient techniques in powering renewable energy to the buildings. The ST-OSCs has the potential to be integrated into building facades, windows, and greenhouses extensively [15]. The characteristic of semi-transparent allows ST-OSCs not only works as a generator but also reduces electricity consumption by letting natural light pass through [15]. In terms of the application in greenhouses, the introduction of ST-OSCs can have little impact on the growth of plants. Since the chlorophylls only function in a small range of the light spectrum, ST-OSCs can be tuned to highly transparent over the spectrum essential for the plant growth while producing power using the rest during the device fabrication [13,16]. For these applications of ST-OSCs, decent transparency under the visible spectrum plays a vital role, and 25% of average visible transmittance (AVT) is a generally acceptable transmittance for window applications [10]. Herein, it is critical to maintain a suitable transmittance along with high power conversion efficiency for ST-OSCs.

For ST-OSCs, the transparent top electrode is an indispensable component, where the D/M/D-structured electrode displayed a promising future [13,17,18,19,20]. The D/M/D electrode has been gradually used to replace ITO in solar cell manufacturing in recent years, and it was designed to increase the conductivity and flexibility of ITO as it introduces ultra-thin metallic films [21]. Compared with the thick reflective metal electrodes, D/M/D structures are relatively simpler to be constructed and do not contain complicated nano-level patterns [11]. As the transparent electrodes inherently have low reflectivity, the photon absorption of the device should be carefully tuned to let sufficient light transmit through the device [18]. Besides, the neutral colour perception is another crucial element for ST-OSCs, which indicates that the light colour should remain similar after passing through the device [22]. The colour characterisation of an inverted semi-transparent solar cell structure is first mentioned in the study in 2010 [14]. By following the CIE instruction, the colour rendering index (CRI) can be calculated with the device transmittance within the visible region [23,24]. CRI is the value to quantify the ability of the light source to display the actual colour of an object compared with a standard source, ranging from 0 to 100. Hence, it can be used as a method to identify the distinction of the transmitted light colour and one of the original lights [25]. In the window application, the CRI value should be as high as possible while the colour coordinates should be close to the Planckian locus [26].

In recent years, many studies have been conducted on non-fullerene based OSCs [27]. Different from common fullerene-based OSCs, non-fullerene-based OSCs show great potential in tunning energy level, absorbing incident light, and tunning molecular structures [28,29]. Recently, Zou’s team synthesised the novel non-fullerene acceptor N3, which was adjusted from the state-of-the-art non-fullerene material Y6 and showed advanced photovoltaic performance [30,31]. Our previous work investigated the stability of PM6:N3-based organic solar cells, where the device was fabricated opaque and tested under burn-in degradation. As a result, the PM6:N3-based active layer was found to be relatively stable and showed a huge potential for achieving future application [32]. However, there is no current study which focuses on the semi-transparent OSCs based on the N3 non-fullerene with D/M/D electrode structure, leaving this gap in the research to be filled. In this work, by using the MoO_3_-/Ag-/MoO_3_-based D/M/D transparent electrodes, we fabricated ST-OSCs based on the Poly[(2,6-(4,8-bis(5-(2-ethylhexyl-3-fluoro)thiophen-2-yl)-benzo[1,2-b:4,5-b′]dithiophene))-alt-(5,5-(1′,3′-di-2-thienyl-5′,7′-bis(2-ethylhexyl)benzo[1′,2′-c:4′,5′-c′]dithiophene-4,8-dione)] (PM6):N3 active layer for the first time. N3 is a small molecule acceptor with third-position branched alkyl chains [30]. In the device fabrication, the D/M/D transparent electrode was optimised by varying the thickness of the outer MoO_3_ layer. As a result, we found that increasing the thickness of the outer MoO_3_ layer can increase the average visible transmittance (AVT) but decrease the power conversion efficiency (PCE) of the device. By varying the outer MoO_3_ layer thickness from 0 to 30 nm, the average PCE of the device decreased from 13.79% to 7.89%, with an increase in the AVT from 24.45% to 31.26%. The outer MoO_3_ layer with 10 nm thickness was found to deliver the optimum case, where its corresponding device showed the PCE of 9.18% with a high AVT of 28.94%. Moreover, by following the CIE protocol, the CRI values were calculated for all ST-OSCs. As a result, all the devices displayed a neutral colour perception with a high CRI value over 90.

## 2. Experimental Details

### 2.1. Materials Preparation

The pre-patterned ITO glasses with the area of 12 × 12 mm^2^ were purchased from Lumtec (Taipei, Taiwan). The chemical materials PM6 was purchased from 1-Materials (Dorval, Canada). The chemical materials N3 was synthesized by Zou’s group. The chemical material, zinc oxide nanoparticles, reagent alcohol (anhydrous, <0.003% water), chlorobenzene (99.8%), 1,8-diiodooctane, and MoO_3_ were purchased from Sigma-Aldrich (Sydney, Australia).

### 2.2. Device Fabrication

In this study, PM6:N3 layer was applied as the active layer in the bulk heterojunction (BHJ) OSCs with the inverted device architecture of ITO glass (0.7 mm)/ZnO(40 nm)/active layer (100 nm)/MoO_3_(10 nm)/Ag(10 nm)/MoO_3_. Firstly, the ITO glass substrate was ultrasonicated in the sequence of soapy deionised (DI) water, pure DI water, acetone and isopropanol to remove the residue on it. Secondly, zinc acetate dihydrate (Zn(CH_3_CO_2_)_2_·2H_2_O, Sigma-Aldrich, Sydney, Australia, >99.0%, 0.109 g) and ethanolamine (NH_2_CH_2_CH_2_OH, Sigma-Aldrich, >99.5%, 32 µL) was dissolved in 2-methoxy ethanol (CH_3_OCH_2_CH_2_OH, Sigma-Aldrich, 99.8%, anhydrous, 1 mL) in the preparation of ZnO sol-gel solution (0.48 M). Then the ZnO sol-gel layer was fabricated on the top of the clean ITO glass substrates through spin casting at 4000 rpm for 1 min and annealed for 30 min at the temperature of 170 °C. Thirdly, 10 mg PM6 and 12 mg N3 with a 1:1.2 wt ratio was mixed in a 17.6 mg/mL chlorobenzene solution added with 0.5% vol 1-Chloronaphthalene (CN) for the preparation of the active layer solutions. Fourthly, the active layer solution was mixed thoroughly by stirring overnight inside an N2-filled glovebox with the temperature maintained at 80 °C and then deposited onto the substrates at a rate of 2000 rpm for 60 s. Afterwards, the coated samples were put in a vacuum chamber under the pressure of 10^−5^ Pa. Lastly, the 10-nm-thick film of MoO_3_ and 100-nm-thick film of silver was deposited onto the sample surface through a shadow mask by thermal evaporation for the opaque device. The 10-nm-thick film of MoO_3_ and 10-nm-thick film of silver and another 0–30-nm-thick film of MoO_3_ was deposited to the sample surface through a shadow mask by thermal evaporation for the semi-transparent device. The fabricated device area was 0.045 cm^2^.

### 2.3. Device Characterisation

The samples are kept in an N_2_-filled glovebox to reduce the impact of degradation ahead of characterisation. The current density-voltage (*J-V*) was measured by a solar cell *I-V* testing system (Keithley 2400 source meter, Armley, UK) and illuminated at 100 mW·cm^−2^ by an AM 1.5 G solar simulator. The device temperature was metered and kept at about 25 °C by a GN1350 50:1 LCD infrared thermometer digital gun. The devices’ optical properties were measured by a UV-VIS-NIR spectrometer (Perkin Elmer-Lambda 950, Sydney, Australia). The colour perception was measured by using UV-VIS-NIR spectrometer with a big mask to stable the samples.

## 3. Results and Discussion

**Figure 1:**
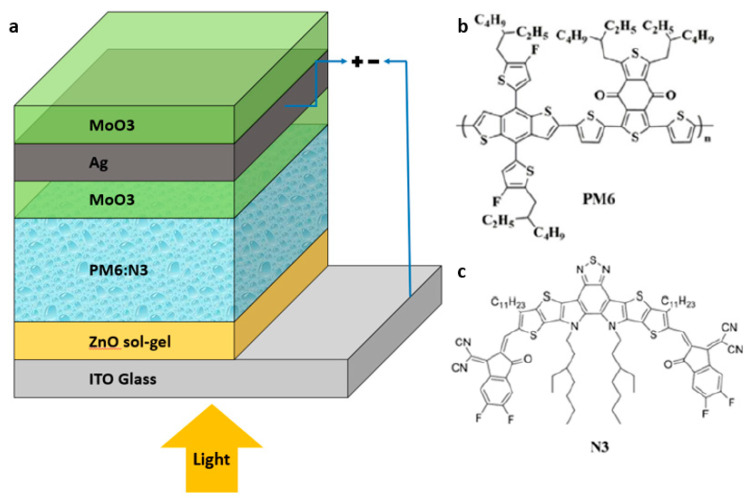
(**a**) The schematic diagram of the inverted device structure of indium tin oxide (ITO)/ZnO/active layer/MoO_3_/Ag/MoO_3_ for the fabricated semi-transparent devices; (**b**) the diagram of chemical structures of PM6; (**c**) the diagram of chemical structures of N3.

The inverted device structure of the PM6:N3-based ST-OSCs (ITO/ZnO/active layer/MoO_3_/Ag/MoO_3_) is displayed in Figure 1. From bottom to top, the ITO works as a see-through electrode. Above the ITO, a 40 nm thickness of the ZnO sol-gel layer works as an electron transport layer (ETL). In the middle of the device, a photoactive layer, which is made of the donor material (PM6) and the acceptor material (N3), forms the bulk heterojunction (BHJ). For the D/M/D-structured electrode, a 10-nm-thick inner MoO_3_ layer operates as the hole transport layer. Sandwiched between the MoO_3_ layers, a 10-nm-thick silver layer runs as the top translucent electrode for the semi-transparent device. The thickness of the outer MoO_3_ layer is optimised from 0 to 30 nm. For the opaque device, the top electrode is commonly used thick silver layer with a thickness of 100 nm [33]. For the semi-transparent devices, the top electrode we used is a MoO_3_-/Ag-/MoO_3_-based D/M/D electrode [17].

**Figure 2:**
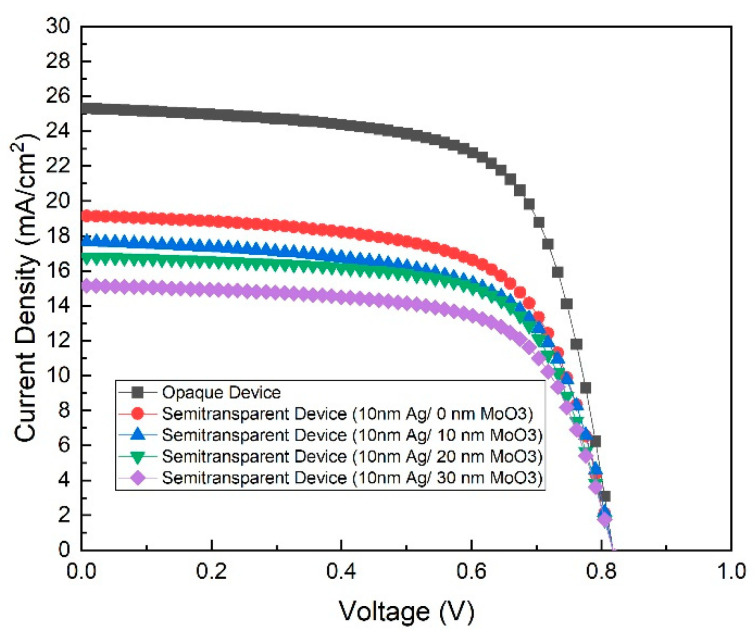
The current density to voltage (*J-V*) curves of the opaque device and all fabricated semi-transparent devices.

Figure 2 shows the current density to voltage (*J-V*) curves for all fabricated devices. The corresponding photovoltaic parameters of the device, including the open-circuit voltage (*V_oc_*), short-circuit current density (*J_sc_*), power conversion efficiency (PCE), series resistance (*R_s_*), and shunt resistance (*R_sh_*) are displayed in Table 1. For all the devices, the *V_oc_* values are around 0.8 V, pointing out that the variation of the Ag thickness and the outer MoO_3_ layer thickness has a negligible effect on the device voltage. The opaque device with 100-nm-thick Ag layer achieved 25.29 mA·cm^−2^ of *J_sc_*, 66.9% of FF and 13.8% of PCE. By reducing the thickness of the Ag layer to 10 nm, *J_sc_* dropped from 24.3% to 19.15 mA cm^−2^, with a 27.8% decrease in PCE. The reduction in *J_sc_* may result from the decrease in the creation and collection of light-generated carriers capacity. The decrease in the photovoltaic performance accompanies with the enhancement in the transparency of the devices [34].

When optimising the thickness of the outer MoO_3_ layer from 0 to 30 mn, a variation of PCE from 9.96% to 7.84 was observed. Overall, when varying the silver layer thickness from 100 to 10 nm, the PCE of the device decreased. The decrease in the absorption might cause a reduction in PCE. Hence, the experiments were designed to compare the transmission and reflection of the devices.

**Figure 3:**
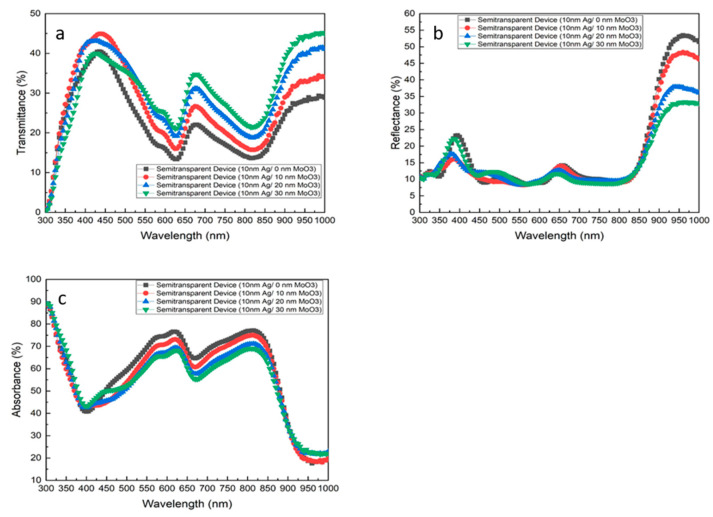
(**a**) Transmittance, (**b**) reflectance and (**c**) absorbance spectra of the semi-transparent devices over different outer MoO_3_ layer thicknesses.

The transmittance, reflectance, and absorbance spectrums of semi-transparent devices were measured and displayed in Figure 3. The measured wavelength ranges from 300 to 1000 nm, covering the visible light spectrum (380 to 780 nm) [33]. The transmittance spectrum of the devices was presented in Figure 3a. The first peak of the transmittance spectrum is observed between the range from 380 to 480 nm. By increasing the thickness of the outer MoO_3_ layer from 0 to 10 nm, there is a 10.2% increase in the transmittance of the device from 40.3% to 44.9%. However, a 4.9% and another 8.0% drop in the device transmittance is observed by increasing the layer thickness to 20 and 30 nm. In the second and the third peak of the transmittance spectrum, 36.6% and 35.4% of increases are observed when the layer thickness increased to 30 nm. Generally, as the thickness of the outer MoO_3_ layer increases, the overall device transmittance increases.

In order to investigate the absorption of the devices, the reflectance spectrum of the devices was measured. The following equation can calculate the absorption of the device:

Absorption = 100 − (Transmission + Reflection)(1)

The reflectance spectrum of the devices is presented in Figure 3b. The highest peak of the reflectance spectrum is observed at around 975 nm of the wavelength, with a value of 53%. The second peak is observed at around 400 nm, where the reflectance achieved about 38%. From 300–600 nm, the reflectance of the device generally increases when the outer MoO_3_ layer increases from 0 to 30 nm. Over 880 nm, around 40% of decrease is observed by increasing the outer MoO_3_ layer thickness from 0 to 30 nm. In general, as the thickness of the outer MoO_3_ layer increases, the device reflectance increases at the wavelength range from 300 to 550 nm and decreases at the wavelength range from 550 to 1000 nm.

With the measured transmittance and reflectance, the device absorption was calculated. The absorbance spectrum of the devices is presented in Figure 3c. It is apparently to see that increasing the outer MoO_3_ layer thickness causes the device absorbance to decrease. The decrease in device absorbance was mainly caused by the increase of transmittance from 480 to 880 nm. By increasing the outer MoO_3_ layer thickness from 0 to 30 nm, around 20% of the decrease in absorbance is observed. Relating to the photovoltaic performance of the devices, a 22% drop from 10.0% of PCE to 7.8% is observed by increasing the outer MoO_3_ layer thickness from 0 to 30 nm. The reduction in PCE may result from the reduction of the light trapping in the device.

The AVT of ST-OSCs evaluates its semitransparency properties. Only visible light in the range of 380–780 nm was taken into consideration to describe the transmittance of the device considering the sensitivity of the human eye. The AVT was calculated by using the following equation [35]:

(2) $$AVT=\frac{\int T\left(\lambda \right)P\left(\lambda \right)S\left(\lambda \right)d\lambda }{\int P\left(\lambda \right)S\left(\lambda \right)d\lambda }$$

where *λ* represents the light wavelength, *S* represents the solar photon flux, *T* is the transmittance, and *P* is the photopic response, which is dependent on the spectral sensitivity of the human eye. A spectrophotometer is commonly used to measure the transmittance of the device [35]. It is worth mentioning that the required AVT value strongly depends on the specific application. For example, a 25% AVT is commonly required for window applications for ST-OSCs in BIPVs [10]. The AVT of different devices was calculated and presented in Table 2. As the light cannot pass through the opaque device, the AVT of the device is 0%. For the semi-transparent devices, by increasing the outer MoO_3_ layer thickness, the AVT increased from 24.45% to 31.26%. In general, when the MoO_3_ layer thickness increased, the device transmission within the visible range increased. However, the device PCE is also an essential factor in commercialisation. Hence, the relationship between AVT and PCE was investigated, and the device was optimised to achieve a balanced result.

**Figure 4:**
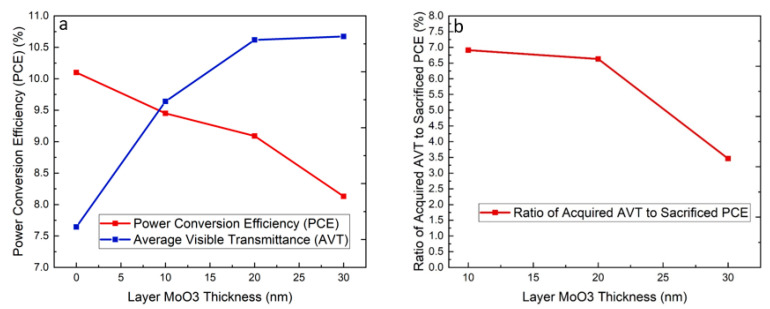
(**a**) The relationship between the power conversion efficiency (PCE) and average transmittance (AVT) versus the outer MoO_3_ layer thickness; (**b**) the ratio of the acquired AVT to sacrificed PCE.

The PCE and AVT of fabricated ST-OSCs are displayed in Figure 4a. From 0 to 10 nm, the AVT increases by 18.36% from 24.45% to 28.94%. By using the 20 nm of the outer MoO_3_ layer thickness, the AVT raises by 7.60% from 28.94% to 31.14%. Moreover, when the thickness of the outer MoO_3_ layer increases to 30 nm, there is only 0.39% increase from 31.14% to 31.26%. However, the increasing trend becomes less apparent when the layer gets thicker. Conversely, the PCE decreases when the outer MoO_3_ layer thickness increases. Increasing the thickness from 0 to 10 nm, the PCE decreases from 9.96% to 9.18%. When the outer MoO_3_ layer thickness increases to 20 nm, the PCE drops from 9.18% to 8.86%. Furthermore, when the thickness of the outer MoO_3_ layer increases to 30 nm, there is an 11.51% decrease in PCE from 8.86% to 7.84%. The ratio of acquired AVT to sacrificed PCE was calculated and displayed in Figure 4b. For the device with 10 nm thickness of the outer MoO_3_ layer thickness, the ratio is 6.91%, indicating 6.91% of AVT can be acquired by sacrificing 1% of PCE. Furthermore, the ratio of 20 and 30 nm is 6.63% and 3.56% equivalently. The result shows that the ratio of the outer MoO_3_ layer with 10 nm thickness is the highest. 28.94% of AVT can be achieved with 9.2% of PCE. Therefore, the device structure with 10 nm of the outer MoO_3_ layer thickness is the optimal case, showing excellent performance in both photovoltaic and optical aspects.

## 4. Colour Rendering Index

The transmission spectrum of the devices can be used to calculate the colour rendering index (CRI), which is a quantified value to describe the ability of a light source to redisplay the actual colour of an object compared with a standard illuminant [18,25,36,37]. CRIs can be calculated by inducing the equations below, which converts the transmittance spectrum in the visible region (from 380 to 780 nm) into a tristimulus system (*X, Y, Z*) and the colour coordinates (*x, y*) [18]:

(3) $$X=\int_{380nm}^{780nm}S\left(\lambda \right)\times \bar{x}\left(\lambda \right)\times T\left(\lambda \right)d\lambda$$

(4) $$\;Y=\int_{380nm}^{780nm}S\left(\lambda \right)\times \bar{y}\left(\lambda \right)\times T\left(\lambda \right)d\lambda$$

(5) $$Z=\int_{380nm}^{780nm}S\left(\lambda \right)\times \bar{z}\left(\lambda \right)\times T\left(\lambda \right)d\lambda$$

(6) $$\;X=\frac{X}{X+Y+Z}$$

(7) $$\;Y=\frac{Y}{X+Y+Z}\;$$

**Figure 5:**
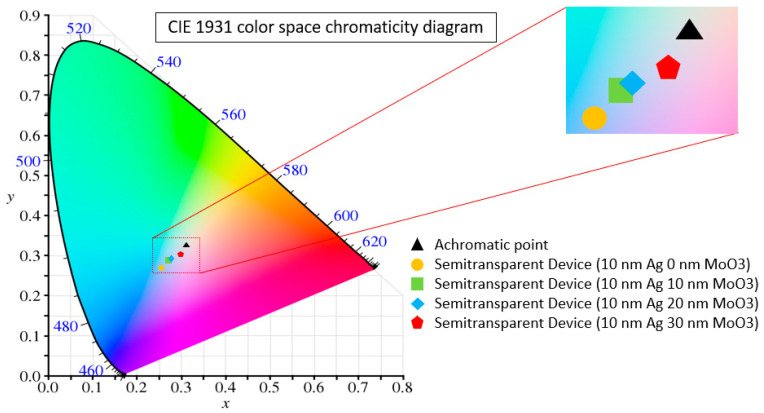
Representation of the colour coordinates (*x, y*) of all devices using a standard D65 illumination light source on the CIE 1931 colour space.

In the Equations (3)–(7), *S*(*λ*) stands for the CIE Standard Illuminant D65, $\bar{x}$, $\bar{y}$, $\bar{z}$ are the colour matching functions defined by the CIE protocol, and the *T*(*λ*) represents the device transmittance spectrum. The coordinates (*x, y*) are the colour coordinates as defined in the CIE 1931 colour space. The colour coordinates display the colour variation of the light perceived by human eyes when transmitting through the semi-transparent devices. The colour coordinates of all devices plotted in the CIE colour space chromaticity diagram, as presented in Figure 5, and the calculated coordinates are displayed in Table 3.

The calculated colour coordinates of the ST devices sit close to the achromatic (white point) on the colour space diagram [38]. It indicates a pleasant colour sensation when the viewers look through cells under the illumination of AM 1.5G. The colour coordinates of the devices with 30 nm thickness of outer MoO_3_ layer are close to the illuminant D65 (0.31, 0.33) at (0.27, 0.30). These devices are capable of letting the light pass through them without affecting much to the original colour of an object. For the devices with a thinner layer thickness (10, 20 nm), the colour coordinates are located near the blue colour on the CIE chromaticity diagram. According to the definition, the CRI equals to the ratio of sample “colour rendering ability” to that of standard source in percentage. It ranges between 0 and 100, the higher the CRI value, the better capability to reveal the original light [10,39]. The CRIs can be calculated by following the CIE 13.3 1995 protocol. The (*x*, *y*) coordinates are converted to (u, v) coordinates in the colour space. The coordinates are adjusted from eight test colour samples, which are known as ref samples and are divided from the visible spectrum, were illuminated under the D65 source. Moreover, the test samples are colours of light which transmitted through the device. The different reflection result of each sample accounts for the change of transmitted light colour [23,24]. As a result, the CRI values of all fabricated ST-OSCs are above 90, and the CRI decreases as the outer MoO_3_ layer thickness increases. Generally, all fabricated PM6:N3-based semi-transparent devices display well neutral-colour perception [33].

## 5. Conclusions

In conclusion, high-performance semi-transparent organic solar cells were fabricated using PM6:N3 as the active layer with a transparent D/M/D (MoO_3_/Ag/MoO_3_) electrode. The thickness of the outer MoO_3_ layer was optimised to balance the trade-off between the PCE and AVT of the device. Our works were conducted to study the relationship between the outer MoO_3_ layer thickness and the device photovoltaic and optical performance. For the opaque device with 100 nm thickness of the silver layer without the outer MoO_3_ layer, the device achieved 25.29 mA cm^−2^ of *J_sc_*, 66.9% of FF and 13.8% of PCE. Compared with the opaque device, the reduction of efficiency in semi-transparent is inevitable due to the reduction of silver layer thickness from 100 to 10 nm, which plays an essential role in light capture ability. Increasing the outer MoO_3_ layer thickness decreased *J_sc_* and PCE, but the device transmittance increased in general. All the devices exhibit excellent optical performance, as the AVTs are all above 24%. By increasing the outer MoO_3_ layer thickness from 0 to 30 nm, AVT increases 23% from 24.45% to 31.26%. We found that the 10 nm thickness of the outer MoO_3_ layer gives the optimum device performance. The corresponding device achieved 19.15 mA·cm^−2^ of *J_sc_*, 64.9% of FF, 10.0% of PCE, 28.94% of AVT and 97 of CRI. The device exhibits excellent PCE and perfect colour rendering property showing great potential for the window application.

## Figures and Tables

**Table 1:** Photovoltaic parameters summary including the Open-Circuit Voltage (*V_oc_*), Short-Circuit Current (*J_sc_*), Fill Factor (FF), Power Conversion Efficiency (PCE), Series Resistance (*R_s_*) and Shunt Resistance (*R_sh_*). The parameters were measured at room temperature under one-sun test condition (AM1.5 G illumination, 100 mW/cm^2^), and the average value and the standard deviation were determined from the measurement of at least five devices.

Devices	*V_oc_* (V)	*J_sc_* (mA/cm^2^)	FF(%)	PCE(%)	*R_s_* (Ω)	*R_sh_* (Ω)
Opaque Device (100 nm Ag/0 nm MoO_3_)	0.82 ± 0.00	25.29 ± 0.71	66.91 ± 1.40	13.79 ± 0.34	131 ± 18	18,444 ± 822
Semi-transparent Device (10 nm Ag/0 nm MoO_3_)	0.80 ± 0.00	19.15 ± 0.31	64.92 ± 1.10	9.96 ± 0.10	144 ± 9	18,511 ± 3111
Semi-transparent Device (10 nm Ag/10 nm MoO_3_)	0.79 ± 0.00	17.68 ± 0.45	65.41 ± 0.45	9.18 ± 0.23	147 ± 4	19,200 ± 1244
Semi-transparent Device (10 nm Ag/20 nm MoO_3_)	0.81 ± 0.00	16.79 ± 0.17	65.46 ± 1.79	8.86 ± 0.20	191 ± 24	25,089 ± 1822
Semi-transparent Device (10 nm Ag/30 nm MoO_3_)	0.80 ± 0.01	15.15 ± 0.53	64.67 ± 2.70	7.84 ± 0.19	207 ± 24	23,333 ± 4933

**Table 2:** The average visible transmittance (AVT) of the opaque and all fabricated semi-transparent devices with different thickness of the outer MoO_3_ layer.

**Devices**	**AVT**
Opaque Device	0.00%
Semitransparent Device (10 nm Ag/0 nm MoO_3_)	24.45%
Semitransparent Device (10 nm Ag/10 nm MoO_3_)	28.94%
Semitransparent Device (10 nm Ag/20 nm MoO_3_)	31.14%
Semitransparent Device (10 nm Ag/30 nm MoO_3_)	31.26%

**Table 3:** Colour rendering index (CRI) and corresponding colour coordinates (*x*, *y*) of different semi-transparent devices.

Devices	*x*	*y*	CRI
Semitransparent Device (10 nm Ag/0 nm MoO_3_)	0.2536	0.2692	97.3
Semitransparent Device (10 nm Ag/10 nm MoO_3_)	0.2702	0.2843	97.0
Semitransparent Device (10 nm Ag/20 nm MoO_3_)	0.2828	0.2942	95.4
Semitransparent Device (10 nm Ag/30 nm MoO_3_)	0.2994	0.3039	92.1
